# State policies regulating short-term limited duration insurance plans and cancer stage at diagnosis

**DOI:** 10.1093/jncics/pkad060

**Published:** 2023-08-12

**Authors:** Justin M Barnes, Anne C Kirchhoff, K Robin Yabroff, Fumiko Chino

**Affiliations:** Department of Radiation Oncology, Washington University School of Medicine in St. Louis, St. Louis, MO, USA; Department of Pediatrics, University of Utah School of Medicine, Salt Lake City, UT, USA; Huntsman Cancer Institute, Salt Lake City, UT, USA; Surveillance and Health Equity Science, American Cancer Society, Atlanta, GA, USA; Department of Radiation Oncology, Memorial Sloan Kettering Cancer Center, New York, NY, USA

## Abstract

Short-term limited duration insurance plans, which proliferated following 2018 federal regulations, may not provide adequate protections for patients with suspected or newly diagnosed cancer and can destabilize insurance markets for comprehensive insurance plan enrollees. Individuals aged 18-64 years with newly diagnosed cancer from 11 states during 2016-2017 and 2019 were identified from the Surveillance, Epidemiology, and End Results program. Difference-in-differences analyses were used to compare changes in early-stage cancer diagnoses from 2016-2017 to 2019 in states that prohibited vs did not regulate short-term limited duration insurance plans. In adjusted difference-in-differences analyses, early-stage diagnoses increased 0.95 percentage points (95% confidence interval = 0.53 to 1.38, *P* < .001) in states that prohibited short-term limited duration insurance plans vs did not regulate short-term limited duration insurance plans. State policies resulting in unavailability of short-term limited duration insurance plans were associated with an increased percentage of early-stage diagnoses.

In 2018, federal regulations expanded access to short-term limited duration insurance plans, which can exclude people with preexisting conditions like cancer, limit coverage of medical services including cancer screening, and have no cost-sharing caps (1,2). Individuals enrolled in short-term limited duration insurance plans may have inadequate access to cancer-related care or catastrophic out-of-pocket expenses, potentially delaying a cancer diagnosis (2). Premiums for short-term limited duration insurance plans are lower than plans with comprehensive coverage with essential health benefits, and in 2019, approximately 3 million Americans were enrolled in short-term limited duration insurance plans (3).

Uptake of short-term limited duration insurance plans by healthy individuals can increase premium costs for comprehensive insurance plan enrollees by changing risk pools and destabilizing markets for individual insurance (2). Several states introduced legislation prohibiting and/or limiting short-term limited duration insurance plans, which stabilized markets and decreased costs for individuals with comprehensive coverage (1,4,5). Our objectives were to examine associations of short-term limited duration insurance plans and associated state-level policies on cancer stage at diagnosis.

Individuals aged 18-64 years with staged first primary malignancies diagnosed 2016-2019 were identified from Surveillance, Epidemiology, and End Results (SEER) registry data from 11 states covering 26.5% of the US population (Supplementary Figure 1, available online). Difference-in-differences analyses evaluated changes in the percentage of early-stage (stages 0 [breast and bladder only] through II) diagnoses pre- (2016-2017) and post–short-term limited duration insurance expansion (2019; 2018 was excluded as washout and phase-in period) between states with policies prohibiting and/or limiting short-term limited duration insurance plans vs states with no policies regulating short-term limited duration insurance plans (Supplementary Table 1, available online). In some states limiting but not prohibiting short-term limited duration insurance plans, insurers stopped selling short-term limited duration insurance plans, prompting additional analyses by state short-term limited duration insurance plan availability in 2019. Linear probability models accounted for state clustering and adjusted for age, race, ethnicity, sex, metropolitan residence status, marital status, county household income, cancer site, and state and year fixed effects.

Plausibility of the parallel trends assumption was evaluated by testing for differential changes in early-stage diagnosis percentages from 2016 to 2017 by state group (Supplementary Table 2, available online) (6). Sensitivity analyses excluded states that expanded and/or substantially changed Medicaid eligibility during the study period. Additional sensitivity analyses explored state-specific policy changes. Subgroup analyses of screening-detectable and other cancers were performed. Falsification tests used a sample of individuals aged 65 years and older, who were Medicare-age eligible and unlikely to be affected by short-term limited duration insurance plans (Supplementary Methods, available online).

A total of 398 190 individuals were included (Supplementary Table 3, available online). Early-stage diagnoses increased 0.95 percentage points (95% confidence interval [CI] = 0.53 to 1.38, *P* < .001) in states that prohibited short-term limited duration insurance plans vs did not regulate short-term limited duration insurance plans, translating to approximately 650 more early-stage diagnoses in 2019 in short-term limited duration insurance–prohibiting states, with less change in states with other short-term limited duration insurance policies (−0.71 percentage points, 95% CI = −1.92 to 0.50, *P* = .25) (Table 1 and Figure 1, A). Relative to states where short-term limited duration insurance plans remained available, there was a 0.77 (95% CI = 0.00 to 1.53, *P* = .05) percentage point increase in early-stage diagnoses in states where legislation stopped the sale of short-term limited duration insurance plans (after excluding states with Medicaid eligibility changes: 1.03 percentage points, 95% CI = 0.71 to 1.35, *P* < .001) (Table 1 and Figure 1, B), translating to more than 800 more early-stage diagnoses. Results were similar for potentially screening-detectable cancers and other cancers (Supplementary Table 4, available online).

**Table 1.** Changes in stage at diagnosis associated with state-level policies addressing expanded access to short-term limited duration health insurance plans

Analysis	State group	Temporal trends in % early stage (stages 0-II)		Difference-in-differences analyses					
				Main analyses				Sensitivity analyses excluding states with Medicaid eligibility changes^a^	
		2016-2017	2019	Unadjusted estimate (95% CI)	*P*	Adjusted estimate (95% CI)	*P*	Adjusted estimate (95% CI)	*P*
State-level short-term limited duration insurance plan policies	No short-term limited duration insurance plan policies^b^	62.6	64.7	Referent		Referent		Referent	
	Some short-term limited duration insurance plan regulation^c^	66.7	68.4	−0.31 (−1.92 to 1.3)	.71	−0.71 (−1.92 to 0.5)	.25	0 (−0.81 to 0.81)	.99
	Prohibit short-term limited duration insurance plans^d^	65.1	67.9	0.73 (0.15 to 1.31)	.013	0.95 (0.53 to 1.38)	<.001	0.93 (0.59 to 1.27)	<.001
State-level short-term limited duration insurance plan availability	Short-term limited duration insurance plans available^e^	63.0	65.1	Referent		Referent		Referent	
	No short-term limited duration insurance plans available in 2019^f^	65.4	68.1	0.46 (−0.36 to 1.28)	.27	0.77 (0.00 to 1.53)	.05	1.03 (0.71 to 1.35)	<.001

a Excluded states include Louisiana (expanded Medicaid in mid-2016), Utah (partial Medicaid expansion in 2019), and Connecticut (multiple Medicaid eligibility changes and/or restrictions introduced from 2015 to 2018). See Supplementary Table 1 (available online) for additional information. CI = confidence interval.

b Georgia, Iowa, Kentucky, and Utah.

c Connecticut, Hawaii, New Mexico, and Washington.

d California and New Jersey.

e Georgia, Iowa, Kentucky, Louisiana, Utah, and Washington.

f California, New Jersey, and Hawaii. Note that New Mexico also introduced policies that ultimately led to insurers to stop offering short-term limited duration insurance plans, but this occurred in mid-2019, so this state was excluded. See Supplementary Table 1 (available online) for additional information.

**Figure 1.**
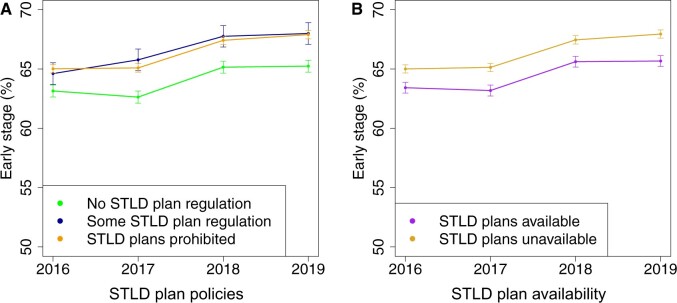
Changes in percentage early-stage diagnoses from 2016 to 2019 by **(A)** state policies restricting short-term limited duration insurance (STLD) plans and **(B)** state STLD plan availability. STLD plan access was expanded nationally by 2018 federal regulation. Although the figure demonstrates estimates for 2018 for visual purposes in portraying trends, 2018 was excluded from the difference-in-difference analyses as a washout and phase-in period. States with no STLD plan regulation included Georgia, Iowa, Kentucky, and Utah; states that introduced legislation regulating STLD plans but still allowed STLD plans included Connecticut, Hawaii, New Mexico, and Washington; states that prohibited STLD plans included California and New Jersey. States with no STLD plan availability in 2019 included California, New Jersey, and Hawaii; states where STLD plans were available included Georgia, Iowa, Kentucky, and Washington; note cases from Connecticut, Louisiana, and Utah were excluded from these comparisons because of Medicaid eligibility changes. Data source: authors’ analysis of Surveillance, Epidemiology, and End Results data.

Relative to states without policies enhancing access to care, states with increases in early-stage diagnosis percentages associated with 2018-2019 health policy legislation included California, Hawaii, New Jersey, and New Mexico (Supplementary Table 5, available online), which each enacted policies leading to cessation of sale of short-term limited duration insurance plans but shared no other contemporary health policies (Supplementary Table 1, available online). There were no statistically significant changes in cancer stage by state short-term limited duration insurance status in falsification tests (Supplementary Table 6, available online).

Using SEER data spanning 11 states, state legislation prohibiting short-term limited duration insurance plans was associated with a differentially increased percentage of early-stage cancer diagnoses, translating to approximately 650-800 more individuals diagnosed with early rather than late-stage cancers. In other words, states allowing the sale of short-term limited duration insurance plans experienced a relative decrease in percentages of early-stage cancer diagnoses. However, limited legislation regulating but still enabling sale of short-term limited duration insurance plans was not associated with changes in the percentage of early-stage diagnoses.

State-level short-term limited duration insurance policies may influence stage at diagnosis in at least 2 ways. First, short-term limited duration insurance plan enrollees may experience coverage gaps and delays for cancer screening and/or diagnostic workup because of inadequate health-care access (2,7). Second, comprehensive insurance plan enrollees in states without short-term limited duration insurance restrictions may experience higher premiums (>4% higher in 1 report) given altered insurance risk pools, as comprehensive plans concentrate older and less healthy enrollees when younger and/or healthier consumers shift to short-term limited duration insurance plans (5). Alterations of the insurance market in short-term limited duration insurance–friendly states could lead to cost-related barriers to care that could delay a cancer diagnosis (2,5).

This is the first study to our knowledge to assess potential impacts of short-term limited duration insurance plans and associated policies on cancer diagnosis. However, the study is limited by its retrospective nature. Many states enacted other policies in the time period that could affect the health insurance market and care delivery and ultimately confound our findings. However, in a sensitivity analysis, all states with statistically significant increases in early-stage diagnoses severely restricted short-term limited duration insurance plans, and those states shared no other contemporary health insurance policies. Additionally, our study was limited to 11 states available within the SEER program, which may not be representative of other states. Our study period is limited, with only 1-year follow-up after short-term limited duration insurance plan expansion, so long-term impacts are unclear. Furthermore, it is unclear whether individuals who otherwise would be uninsured might benefit from short-term limited duration insurance plans, though the difference-in-differences results suggest a net harm. Finally, individual insurance plan data are lacking; however, to the best of our knowledge, there are no existing databases that adequately capture short-term limited duration insurance plan information, so a quasi-experimental analysis such as this may provide the best evidence regarding the clinical impacts of short-term limited duration insurance plans.

In conclusion, state policies prohibiting short-term limited duration insurance plans were associated with increased percentages of early-stage diagnoses. Our findings can inform state and federal policy makers about the adverse and unintended consequences of short-term limited duration insurance plans as part of efforts to improve cancer outcomes, particularly in the context of a new proposed federal rule limiting short-term limited duration insurance plans (8).

## Supplementary Material

pkad060_Supplementary_Data

## Data Availability

No new data were analyzed in support of this research. The data analyzed included data from the Surveillance, Epidemiology, and End Results program, which can be obtained upon request from the National Cancer Institute (https://seer.cancer.gov/data/access.html).

## References

[1] Giovannelli J , Lucia K, Corlette S. What is your state doing to affect access to health insurance. Commonwealth Fund; 2021. https://www.commonwealthfund.org/publications/maps-and-interactives/2021/oct/what-your-state-doing-affect-access-adequate-health?redirect_source=/publications/maps-and-interactives/2019/sep/what-your-state-doing-affect-access-adequate-health. Accessed October 10, 2022.

[2] Barnes JM , Chino F. Short-term health insurance plans come up short for patients with cancer. JAMA Oncol. 2022;8(8):1101-1103. doi:10.1001/jamaoncol.2022.1624. 35653126

[3] Pallone F , Eshoo AG, DeGette D. Shortchanged: How the Trump Administration’s Expansion of Junk Short-Term Health Insurance Plans Is Putting Americans at Risk. US House of Representatives; 2020. https://docs.house.gov/meetings/IF/IF14/20210323/111378/HHRG-117-IF14-20210323-SD023.pdf. Accessed June 14, 2021.

[4] Norris L. Short-term health insurance availability in your state. Healthinsurance.org; 2021. https://www.healthinsurance.org/short-term-health-insurance/. Accessed October 10, 2022.

[5] Hansen D , Dieguez G. The impact of short-term limited-duration policy expansion on patients and the ACA individual market an analysis of the STLD policy expansion and other regulatory actions on patient spending, premiums, and enrollment in the ACA individual market. Milliman Research Report; 2020. https://www.milliman.com/en/insight/the-impact-of-short-term-limited-duration-policy-expansion-on-patients-and-the-aca-individual-market. Accessed June 14, 2021.

[6] Dimick JB , Ryan AM. Methods for evaluating changes in health care policy: the difference-in-differences approach. JAMA. 2014;312(22):2401-2402. doi:10.1001/jama.2014. 25490331

[7] Partnership to Protect Coverage. *UNDER-COVERED: How “Insurance-Like” Products Are Leaving Patients Exposed*. 2021. https://www.lls.org/sites/default/files/National/undercovered_report.pdf. Accessed June 14, 2021.

[8] Internal Revenue Service, Department of the Treasury, Employee Benefits Security Administration, Department of Labor, Centers for Medicare & Medicaid Services, Department of Health and Human Services. Short-term, limited-duration insurance; independent, noncoordinated excepted benefits coverage; level-funded plan arrangements; and tax treatment of certain accident and health insurance. https://www.federalregister.gov/documents/2023/07/12/2023-14238/short-term-limited-duration-insurance-independent-noncoordinated-excepted-benefits-coverage. Published 2023. Accessed July 26, 2023.

