# Supplementary material for: State policies regulating short-term limited duration insurance plans and cancer stage at diagnosis
Source: JNCI Cancer Spectr. 2023 Aug 12;7(5):pkad060. doi: 10.1093/jncics/pkad060 (PMC10471520; doi:10.1093/jncics/pkad060)
Supplement: pkad060_Supplementary_Data [file pkad060_supplementary_data.pdf]

State policies regulating short-term limited duration insurance plans and cancer stage at diagnosis

SUPPLEMENTARY METHODS

In this study, we treat 2018 as a washout/phase-in period since this was the year most legislation surrounding short-term limited duration insurance (STLD) plans was passed. We considered including it as part of the pre-legislation period since most individuals would not be subject to potential insurance coverage changes until the open enrollment period with start date effective the beginning of the next calendar year, but there were issues with non-parallel trends between states and state groups in 2018 for several analyses (data not shown).

Note all statistical tests were 2-sided, and we considered $P<.05$ statistically significant.

Definition of early-stage diagnosis

A cancer stage (combined group stage) of I-II based on the combined stage group was considered an early-stage diagnosis. Stage 0 cancers were excluded except for cancers of the breast and bladder, where stage 0 cancers were also considered early-stage diagnoses. For our primary analyses, we excluded cases with unstaged cancers, as many of these have no defined stage (e.g. central nervous system [CNS] tumors and leukemia/myeloma; see Figure S1).

As a sensitivity analysis, we did not exclude non-CNS/non-leukemia/non-myeloma cases with missing stage at diagnosis (i.e., CNS, leukemia, and myeloma cases were still excluded), and we

utilized the summary stage (i.e. in situ, localized, regional, or distant) variable, which had less missing information. For the sensitivity analysis, in situ (breast and bladder only) and localized cancers were considered early stage, and all others, including unknown/unstaged tumors, were considered not early stage. In adjusted analyses, after excluding states with adjustment of Medicaid eligibility during the study period, there was a significant increase in early-stage diagnoses in 2019 in states prohibiting STLD plans relative to states with no STLD plan policies (0.18 percentage points, 95% CI = 0.08 to 0.28, P=.001), and there was a nonsignificant increase in early-stage diagnoses in states with no STLD plans available relative to states where STLD plans were available (0.42 percentage points, 95% CI = -0.1 to 0.93, P=.112). These estimates are somewhat attenuated relative to the main manuscript findings, but generally consistent with the overall conclusions.

Note that we focus on the percentage of early-stage diagnoses to identify shifts in stage at diagnosis. A similar yet separate endpoint includes the rate of early-stage cancer diagnoses (i.e., number of new cancer diagnoses per population). However, such an analysis requires different data than available in the present study.

Parallel trends assumption

The parallel trends assumption was assessed during 2016-2017 by examining the interaction term between year and state group by STLD policies in linear probability models with clustered standard errors, adjusted for the same covariates in the main analyses. There were no significant interactions for our main analyses or sensitivity analyses, suggesting the assumption

of parallel trends is reasonable. Note that there were potential violations of the assumption in our falsification tests limiting the sample to age ≥65 years.

Sensitivity analysis of state-by-state changes

While we focus on STLD plans in our primary analyses, there were a number of other policies enacted seeking to stabilize the health insurance market and improve access to care for individuals during the study period (see Supplementary Table 1). We created a separate difference-in-differences model evaluating changes in early-stage cancer diagnoses from 2016-2017 to 2019 in states that did not enact any policy protecting access to care and/or the health insurance market (Iowa and Kentucky; due to issues with non-parallel trends between state groups pre-2018 and due to Medicaid expansions in later years, Louisiana and Utah were excluded; see eTable 1) compared to the other states individually.

Subgroup analyses for screening-detectable cancers

Screening-detectable cancers were defined as female breast cancer diagnosed from age 40-64 years, female cervical cancer diagnosed from age 21-64 years, or colorectal cancer diagnosed from age 45-64 years (note upper limit of 64 years is due to study inclusion criteria). Other cancers include cases of cancer of other sites as well as breast, cervical, or colorectal cancer diagnosed prior to the recommended screening age for average-risk individuals. The same statistical analyses utilized in the main analyses were then applied to these subgroups separately. To formally test whether there were differential effects of STLD policies on early stage diagnosis by screening-detectable cancers, our regression models were expanded to

include a three-way interaction term between DID term (already an interaction term) and an indicator for a cancer that was screening-detectable.

Falsification test

We also conducted a falsification test, where we evaluated changes in early-stage diagnoses from 2016-2017 to 2019 between the state groups among individuals who were expected to be relatively unaffected by STLD plans or associated policies. In this case, we evaluated early-stage diagnoses in individuals ≥65 years, as they are Medicare-age eligible and unlikely to enroll in STLD plans. Other than the different cohort of individuals, the methods were identical to the main analyses.

ADDITIONAL NOTES

A careful reader will notice that, for the main analyses of 18-64 year old patients with cancer, the percentage of early stage diagnoses increased over time for all state groups and was higher for states that enacted some form of legislation limiting STLD plans. The increase in the percentage of early-stage diagnoses over time likely reflects a continuation of 'global' trends of a shift toward earlier stage diagnoses among non-elderly patients with cancer over the past several years, perhaps due to efforts to improve access to care nationwide. For example, Han et al. in a study of Medicaid expansion and non-expansion states demonstrated increases in early-stage diagnoses over the period of 2010-2014 in both groups of states.[1] Differences in baseline rates of early-stage diagnoses by state groups is also well-documented in the Medicaid

expansion literature and likely reflects differing risk factors (e.g. smoking and obesity rates) as well as differences in access to care due to social determinants of health (e.g. insurance rates, poverty, rural populations, etc.), some of which likely are related to state legislation and priorities.[1] In the context of potential state-level differences in stage at diagnosis based on insurance-related factors, the lack of differences by state or changes over time for the 65 years and older age group (see falsification test results, Supplementary Table 6) are not surprising since these individuals are Medicare-age-eligible and thereby less impacted by state-level insurance policies.

References

1. Han X, Yabroff KR, Ward E, Brawley OW, Jemal A. Comparison of Insurance Status and Diagnosis Stage Among Patients With Newly Diagnosed Cancer Before vs After Implementation of the Patient Protection and Affordable Care Act. *JAMA Oncol*. Published online 2018. doi:10.1001/JAMAONCOL.2018.3467

Supplementary Table 1: State policies affecting short-term limited duration insurance (STLD) plans and other policies affecting the health insurance market in SEER registry states

	STLD				Individuals required to maintain adequate health coverage	Reinsurance	Coverage Subsidies	Standardized individual market health plans[l]	Extended Open Enrollment Period for Marketplace Coverage	Allows noncompliant transitional coverage policies[m]	Farm Bureau coverage exemptions[n]	Medicaid eligibility level changes
	No STLD plan regulations	State legislation limits STLD plan duration	State requirements led insurers to stop offering STLD plans	State legislation prohibits STLD plans								
California				X	X[g]		X[j]	X	X			
Connecticut			X[d]				X[k]	X	X			X[o]
Georgia	X									X		
Hawaii			X[e]							X		
Iowa	X[a]									X	X	
Kentucky	X									X		
Louisiana	X[b]									X		X[p]
New Jersey				X	X[h]	X[i]				X		
New Mexico			X[f]									
Utah	X									X		X[q]
Washington		X[c]						X				

[a]New STLD requirements were introduced in 2020

[b]While duration and renewals of STLD plans follow federal legislation, state law limits STLD plan duration to 6 months total duration if the insurer looks back more than 12 months to determine pre-existing conditions, a feature of most plans.

[c]STLD plans are limited to 3 months duration. Also note that STLD plans were unavailable January-March 2019 due to temporary legislation, but sales resumed in April 2019.

[d]Effective January 2019, state law required STLD plans to cover essential health benefits. As such no insurer sells STLD plans.

[e]State law included strict STLD plan eligibility requirements such that only people who were not eligible to purchase a plan in the exchange in the prior year were eligible. STLD plans have not been available since 2018.

[f]State legislation was introduced in 2019 that strictly regulated sale of STLD plans. STLD plans have not been available since mid-2019.

[g]Effective 2020
[h]Effective 2019
[i]Reinsurance offsets costs to insurers (not consumers) for enrollees with high medical expenses. New Jersey's reinsurance program began in 2019.
[j]In addition to federal subsidies to offset cost sharing for lower income individuals enrolled in Marketplace plans, state provides premium assistance for marketplace enrollees with income < 600% federal poverty level.
[k]In addition to federal subsidies to offset cost sharing for lower income individuals enrolled in Marketplace plans, state provides premium assistance for marketplace enrollees with income < 175% federal poverty level.
[l]These states introduced legislation requiring participating insurers of the individual market standardize certain cost-sharing parameters (e.g. uniform deductibles or co-payments for certain services).
[m]These states introduced legislation exempting certain health coverage products (excluding STLD plans, discussed separately) from traditional insurance requirements introduced under the Affordable Care Act, which may lead to increased prices for comprehensive coverage plans.
[n]State legislation exempts Farm Bureau coverage plans from the definition of health insurance and from health insurance regulations.
[o]Connecticut expanded Medicaid eligibility levels under the ACA. However, there were multiple changes to Medicaid eligibility during the study period (2016-2019), which included stricter eligibility requirements expected to affect 14,000 enrollees in 2015-2016 followed by additional policies in 2017-2018 further shifting Medicaid eligibility levels that may have caused churning for Medicaid enrollees in 2018.
[p]Louisiana expanded Medicaid under the Affordable Care Act in July 2016.
[q]While Utah fully expanded Medicaid under the Affordable Care Act in January 2020, there was a smaller-scale expansion that occurred in April 2019 via a series of Section 1115 waivers. Under the more limited expansion, individuals with income up to 100% federal poverty level could qualify for Medicaid; eligibility levels were increased in January 2020 to 138% federal poverty level.
Data sources: Giovannelli J, Lucia K, Corlette S. What Is Your State Doing to Affect Access to Health Insurance | Commonwealth Fund; 2021. Norris L. Short-Term Health Insurance Availability in Your State; 2021. Accessed October 10, 2022. https://www.healthinsurance.org/short-term-health-insurance/. Norris L. A state-by-state guide to Medicaid expansion, eligibility, enrollment and benefits; 2021. Accessed October 10, 2022. https://www.healthinsurance.org/medicaid/.

Supplementary Table 2: Assessment of the parallel trends assumption: differential changes in early-stage cancer diagnoses by state groups from 2016 to 2017.

Analysis	State group	Main Analyses		Sensitivity analyses excluding states with Medicaid eligibility changes[a]	
		Adjusted estimate (95% CI)	P-value	Adjusted estimate (95% CI)	P-value
State-level STLD plan policies	No STLD plan policies[b]	Reference		Reference	
	Some STLD plan regulation[c]	0.54 (-0.45, 1.53)	0.286	0.68 (-0.64, 1.99)	0.313
	Prohibit STLD plans[d]	-0.03 (-0.91, 0.84)	0.939	-0.05 (-1.15, 1.05)	0.931
State-level STLD plan availability	STLD plans available[e]	Reference		Reference	
	No STLD plans available in 2019[f]	-0.17 (-0.97, 0.64)	0.682	-0.26 (-1.27, 0.74)	0.609

[a]Excluded states include Louisiana (expanded Medicaid in mid-2016), Utah (partial Medicaid expansion in 2019), and Connecticut (multiple Medicaid eligibility changes/restrictions introduced from 2015-2018). See Supplementary Table 1 for additional information.

[b]Georgia, Iowa, Kentucky, and Utah.

[c]Connecticut, Hawaii, New Mexico, and Washington.

[d]California and New Jersey.

[e]Georgia, Iowa, Kentucky, Louisiana, Utah, and Washington.

[f]California, New Jersey, and Hawaii. Note that New Mexico also introduced policies that ultimately led to insurers to stop offering STLD plans, but this occurred in mid-2019, so this state was excluded. See Supplementary Table 1 for additional information.

Supplementary Table 3: Characteristics of the study population, No. (%)

Subgroup		Total	STLD Plan Regulations						STLD Plan Availability in 2019			
			No regulations[a]		Some limitations[b]		Plans prohibited[c]		Available[d]		Unavailable[e]	
			2016-17	2019	2016-17	2019	2016-17	2019	2016-17	2019	2016-17	2019
Age	18-39	44647 (11.2)	9236 (10.6)	4596 (10.6)	4109 (10.4)	2098 (11.2)	16485 (11.7)	8123 (11.9)	10988 (10.6)	5564 (10.9)	18842 (11.5)	9253 (11.7)
	40-54	140160 (35.2)	29897 (34.4)	14588 (33.6)	14051 (35.7)	6440 (34.4)	51096 (36.2)	24088 (35.2)	35659 (34.6)	17260 (33.7)	59385 (36.1)	27856 (35.2)
	55-64	213383 (53.6)	47884 (55)	24206 (55.8)	21220 (53.9)	10209 (54.5)	73733 (52.2)	36131 (52.9)	56549 (54.8)	28408 (55.4)	86288 (52.4)	42138 (53.2)
Race and ethnicity	Non-Hispanic White	243564 (61.2)	63764 (73.3)	31319 (72.2)	26194 (66.5)	12077 (64.4)	75469 (53.4)	34741 (50.8)	76258 (73.9)	37199 (72.6)	89169 (54.2)	40938 (51.7)
	Non-Hispanic Black	48061 (12.1)	18130 (20.8)	9111 (21)	2451 (6.2)	1153 (6.2)	11576 (8.2)	5640 (8.3)	18935 (18.3)	9523 (18.6)	13222 (8)	6381 (8.1)
	Non-Hispanic American Indian/Alaska Native	2264 (0.6)	177 (0.2)	95 (0.2)	660 (1.7)	284 (1.5)	708 (0.5)	340 (0.5)	445 (0.4)	222 (0.4)	1100 (0.7)	497 (0.6)
	Non-Hispanic Asian or Pacific Islander	37347 (9.4)	1565 (1.8)	874 (2)	5119 (13)	2591 (13.8)	18103 (12.8)	9095 (13.3)	3260 (3.2)	1773 (3.5)	21527 (13.1)	10787 (13.6)
	Non-Hispanic Unknown Race	2690 (0.7)	146 (0.2)	154 (0.4)	270 (0.7)	158 (0.8)	1199 (0.8)	763 (1.1)	231 (0.2)	219 (0.4)	1384 (0.8)	856 (1.1)
	Hispanic (All Races)	64264 (16.1)	3235 (3.7)	1837 (4.2)	4686 (11.9)	2484 (13.3)	34259 (24.2)	17763 (26)	4067 (3.9)	2296 (4.5)	38113 (23.2)	19788 (25)
Sex	Male	169170 (42.5)	39282 (45.1)	19588 (45.1)	16555 (42)	7804 (41.6)	57764 (40.9)	28177 (41.2)	46071 (44.6)	22790 (44.5)	67530 (41)	32779 (41.4)
	Female	229020 (57.5)	47735 (54.9)	23802 (54.9)	22825 (58)	10943 (58.4)	83550 (59.1)	40165 (58.8)	57125 (55.4)	28442 (55.5)	96985 (59)	46468 (58.6)
Residence	Non-metropolitan	43182 (10.8)	22449 (25.8)	11060 (25.5)	4003 (10.2)	1903 (10.2)	2586 (1.8)	1181 (1.7)	23634 (22.9)	11575 (22.6)	5404 (3.3)	2569 (3.2)
	Metropolitan	355008 (89.2)	64568 (74.2)	32330 (74.5)	35377 (89.8)	16844 (89.8)	138728 (98.2)	67161 (98.3)	79562 (77.1)	39657 (77.4)	159111 (96.7)	76678 (96.8)
Marital status	Not married	158218 (39.7)	35208 (40.5)	17681 (40.7)	15543 (39.5)	7307 (39)	55618 (39.4)	26861 (39.3)	41282 (40)	20563 (40.1)	65087 (39.6)	31286 (39.5)
	Married	239972 (60.3)	51809 (59.5)	25709 (59.3)	23837 (60.5)	11440 (61)	85696 (60.6)	41481 (60.7)	61914 (60)	30669 (59.9)	99428 (60.4)	47961 (60.5)

County income quartile	County income Q1	82129 (20.6)	40757 (46.8)	19530 (45)	4323 (11)	2016 (10.8)	10736 (7.6)	4767 (7)	41247 (40)	19715 (38.5)	14569 (8.9)	6598 (8.3)
	County income Q2	57372 (14.4)	21065 (24.2)	10922 (25.2)	2730 (6.9)	1224 (6.5)	14274 (10.1)	7157 (10.5)	22388 (21.7)	11437 (22.3)	15681 (9.5)	7866 (9.9)
	County income Q3	114039 (28.6)	17161 (19.7)	8662 (20)	11052 (28.1)	4289 (22.9)	48890 (34.6)	23985 (35.1)	21723 (21.1)	10699 (20.9)	55380 (33.7)	26237 (33.1)
	County income Q4	144650 (36.3)	8034 (9.2)	4276 (9.9)	21275 (54)	11218 (59.8)	67414 (47.7)	32433 (47.5)	17838 (17.3)	9381 (18.3)	78885 (48)	38546 (48.6)
Cancer site	Breast	103780 (26.1)	20514 (23.6)	9967 (23)	10843 (27.5)	5158 (27.5)	39141 (27.7)	18157 (26.6)	24989 (24.2)	12150 (23.7)	45509 (27.7)	21132 (26.7)
	Cervical	6592 (1.7)	1417 (1.6)	753 (1.7)	519 (1.3)	283 (1.5)	2438 (1.7)	1182 (1.7)	1632 (1.6)	876 (1.7)	2742 (1.7)	1342 (1.7)
	Colorectal	33536 (8.4)	7411 (8.5)	3719 (8.6)	3200 (8.1)	1473 (7.9)	11786 (8.3)	5947 (8.7)	8704 (8.4)	4347 (8.5)	13693 (8.3)	6792 (8.6)
	Head and Neck	15612 (3.9)	3993 (4.6)	1889 (4.4)	1622 (4.1)	706 (3.8)	5129 (3.6)	2273 (3.3)	4702 (4.6)	2194 (4.3)	6042 (3.7)	2674 (3.4)
	Hodgkin Lymphoma	3971 (1)	829 (1)	436 (1)	361 (0.9)	180 (1)	1424 (1)	741 (1.1)	992 (1)	502 (1)	1622 (1)	855 (1.1)
	Kidney and Renal Pelvis	17806 (4.5)	4214 (4.8)	2168 (5)	1582 (4)	795 (4.2)	5845 (4.1)	3202 (4.7)	4835 (4.7)	2469 (4.8)	6806 (4.1)	3696 (4.7)
	Liver	7195 (1.8)	1522 (1.7)	711 (1.6)	753 (1.9)	259 (1.4)	2764 (2)	1186 (1.7)	1878 (1.8)	828 (1.6)	3161 (1.9)	1328 (1.7)
	Lung and Bronchus	31086 (7.8)	9465 (10.9)	4387 (10.1)	3069 (7.8)	1302 (6.9)	8908 (6.3)	3955 (5.8)	10831 (10.5)	4940 (9.6)	10611 (6.4)	4704 (5.9)
	Non-Hodgkin Lymphoma	15346 (3.9)	3290 (3.8)	1451 (3.3)	1581 (4)	641 (3.4)	5768 (4.1)	2615 (3.8)	3974 (3.9)	1738 (3.4)	6665 (4.1)	2969 (3.7)
	Ovary	6352 (1.6)	1149 (1.3)	550 (1.3)	534 (1.4)	269 (1.4)	2589 (1.8)	1261 (1.8)	1364 (1.3)	655 (1.3)	2908 (1.8)	1425 (1.8)
	Pancreas	9458 (2.4)	2006 (2.3)	1017 (2.3)	919 (2.3)	421 (2.2)	3437 (2.4)	1658 (2.4)	2380 (2.3)	1198 (2.3)	3982 (2.4)	1898 (2.4)
	Prostate	45211 (11.4)	10431 (12)	5551 (12.8)	4360 (11.1)	2238 (11.9)	14953 (10.6)	7678 (11.2)	12124 (11.7)	6420 (12.5)	17620 (10.7)	9047 (11.4)
	Skin	17271 (4.3)	3862 (4.4)	1953 (4.5)	1720 (4.4)	757 (4)	6244 (4.4)	2735 (4)	4672 (4.5)	2331 (4.5)	7154 (4.3)	3114 (3.9)
	Stomach	5683 (1.4)	1032 (1.2)	498 (1.1)	550 (1.4)	231 (1.2)	2309 (1.6)	1063 (1.6)	1227 (1.2)	573 (1.1)	2664 (1.6)	1219 (1.5)

	Testis	5544 (1.4)	921 (1.1)	514 (1.2)	511 (1.3)	262 (1.4)	2150 (1.5)	1186 (1.7)	1119 (1.1)	624 (1.2)	2463 (1.5)	1338 (1.7)
	Thyroid	23003 (5.8)	4284 (4.9)	2229 (5.1)	2306 (5.9)	1146 (6.1)	8614 (6.1)	4424 (6.5)	5110 (5)	2658 (5.2)	10094 (6.1)	5141 (6.5)
	Urinary Bladder	9652 (2.4)	2175 (2.5)	1048 (2.4)	1083 (2.8)	498 (2.7)	3311 (2.3)	1537 (2.2)	2620 (2.5)	1264 (2.5)	3949 (2.4)	1819 (2.3)
	Uterus	18847 (4.7)	3564 (4.1)	1857 (4.3)	1768 (4.5)	1001 (5.3)	6891 (4.9)	3766 (5.5)	4216 (4.1)	2267 (4.4)	8007 (4.9)	4357 (5.5)
	Other	22245 (5.6)	4938 (5.7)	2692 (6.2)	2099 (5.3)	1127 (6)	7613 (5.4)	3776 (5.5)	5827 (5.6)	3198 (6.2)	8823 (5.4)	4397 (5.5)
% Early-stage diagnosis												
Cancer site	Breast		86.5	88.5	89.2	90.4	86.2	89.4	87.1	88.9	86.7	89.7
	Cervical		62.8	76.1	64.3	76.7	60.3	76.2	62.3	76.6	60.9	75.9
	Colorectal		42.6	45	45	44.8	42.8	44.4	42.6	44.5	43.5	44.8
	Head and Neck		28.7	51.9	30.3	57.6	29.1	56	29	52.5	29.3	56.4
	Hodgkin Lymphoma		55	56.4	61.2	50.6	54.4	55.1	55.8	55.4	55.1	54.4
	Kidney and Renal Pelvis		71.2	71.5	68	72	69.5	69.8	70.5	71.5	69.8	70.1
	Liver		54	58	63.2	57.9	58.9	65	56.7	57.6	59	64.6
	Lung and Bronchus		23.5	25.7	27.8	29	23.9	25.5	24	26	24.7	26.3
	Non-Hodgkin Lymphoma		39.5	40.7	48.6	43.2	45.6	42.4	40.6	40.6	46.1	42.8
	Ovary		42.5	42.2	45.7	49.8	40.7	45.3	42.5	42.6	41.3	46
	Pancreas		36.9	29.6	39.6	31.1	34.6	31.5	37.6	29.6	35.1	31.4
	Prostate		76	69	74.1	67	73.7	66.9	75.1	68.4	74.2	67.2
	Skin		82	81.9	83.6	81.5	83.7	82.6	82.1	81.9	83.9	82.4
	Stomach		34.5	37.1	33.1	39.8	31	37.1	33.3	37.4	31.7	37.4
	Testis		83.4	83.1	86.5	86.6	82.8	83.6	83.4	83.8	83.5	83.8
	Thyroid		83.9	97.8	81.4	98.2	78.4	97.7	83.6	97.9	78.8	97.7
	Urinary Bladder		84.1	85.1	85.7	86.3	85.2	86.7	83.8	85.5	85.7	86.6
	Uterus		78.9	80.8	78.8	81.2	78.8	79.4	78.6	81.2	78.9	79.5
	Other		46.1	40.3	44.2	36.6	45.8	41.5	45.6	39.6	45.7	41

[a]Georgia, Iowa, Kentucky, and Utah.

[b]Connecticut, Hawaii, New Mexico, and Washington.
[c]California and New Jersey.
[d]Georgia, Iowa, Kentucky, Louisiana, Utah, and Washington.
[e]California, New Jersey, and Hawaii. Note that New Mexico also introduced policies that ultimately led to insurers to stop offering STLD plans, but this occurred in mid-2019, so this state was excluded. See Supplementary Table 1 for additional information.

Supplementary Table 4: Subgroup analyses by screening-detectable cancer status.

Subgroup	State Group	Temporal trends in % early stage (stages 0-II)		Assessment of the parallel trends assumption				Adjusted Analyses				Interaction between STLD policy effect (DID) and screening-detectable
		2016-2017	2019	Main Analyses		Excluding states with Medicaid eligibility changes[a]		Main Analyses		Excluding states with Medicaid eligibility changes[a]		
				Estimate (95% CI)	P-value	Estimate[a] (95% CI)	P-value	Estimate (95% CI)	P-value	Estimate[a] (95% CI)	P-value	P_{main} ($P_{exclude\ Medicaid\ changes}$)
Screening-detectable cancers	No STLD plan policies[b]	74.61	77.02	Reference		Reference		Reference		Reference		Reference
	Some STLD plan regulation[c]	79.05	80.73	-0.65 (-1.41, 0.1)	0.09	-0.36 (-1.3, 0.59)	0.459	-1.54 (-2.99, -0.08)	0.039	-0.37 (-1.72, 0.97)	0.586	0.11 (0.36)
	Prohibit STLD plans[d]	75.89	78.85	-0.05 (-0.56, 0.45)	0.836	0.07 (-0.55, 0.7)	0.815	0.74 (0.11, 1.37)	0.022	1.15 (1.06, 1.23)	0	0.80 (0.14)
	STLD plans available[e]	75.35	77.61	Reference		Reference		Reference		Reference		Reference
	No STLD plans available in 2019[f]	76.5	79.23	-0.1 (-0.59, 0.39)	0.691	0.08 (-0.45, 0.61)	0.761	0.72 (-0.28, 1.71)	0.159	1.42 (0.91, 1.93)	0	0.93 (0.075)
Other cancers	No STLD plan policies[b]	57.28	59.39	Reference		Reference		Reference		Reference		Reference
	Some STLD plan regulation[c]	60.33	62.31	1.29 (0.11, 2.46)	0.032	1.4 (-0.15, 2.95)	0.076	-0.4 (-1.69, 0.9)	0.55	0.1 (-0.54, 0.74)	0.759	
	Prohibit STLD plans[d]	59.43	62.42	0.02 (-1.06, 1.09)	0.973	-0.02 (-1.39, 1.35)	0.974	0.93 (0.19, 1.67)	0.014	0.71 (0.18, 1.25)	0.009	
	STLD plans available[e]	57.4	59.64	Reference		Reference		Reference		Reference		

	No STLD plans available in 2019[f]	59.76	62.52	-0.15 (-1.23, 0.92)	0.778	-0.37 (-1.71, 0.97)	0.589	0.7 (-0.2, 1.61)	0.127	0.76 (0.29, 1.22)	0.001	

[a]Excluded states include Louisiana (expanded Medicaid in mid-2016), Utah (partial Medicaid expansion in 2019), and Connecticut (multiple Medicaid eligibility changes/restrictions introduced from 2015-2018). See Supplementary Table 1 for additional information.

[b]Georgia, Iowa, Kentucky, and Utah.

[c]Connecticut, Hawaii, New Mexico, and Washington

[d]California and New Jersey.

[e]Georgia, Iowa, Kentucky, Louisiana, Utah, and Washington.

[f]California, New Jersey, and Hawaii. Note that New Mexico also introduced policies that ultimately led to insurers to stop offering STLD plans, but this occurred in mid-2019, so this state was excluded. See Supplementary Table 1 for additional information.

Supplementary Table 5: Changes in early-stage diagnoses for individual states from 2016-2017 to 2019 relative to states without contemporary health insurance legislation

	Assessment of the parallel trends assumption		Main Analyses	
	Adjusted estimate (95% CI)	P-value	Adjusted estimate (95% CI)	P-value
Iowa	Reference		Reference	
Kentucky				
California	0.59 (-1.08, 2.25)	0.489	0.77 (0.73, 0.81)	<.001
Connecticut	0.66 (-1, 2.32)	0.434	-2.37 (-2.44, -2.31)	<.001
Georgia	0.78 (-0.87, 2.44)	0.354	-0.43 (-0.46, -0.41)	<.001
Hawaii	1.14 (-0.53, 2.81)	0.18	0.88 (0.84, 0.92)	<.001
New Jersey	-0.4 (-2.07, 1.27)	0.638	0.42 (0.37, 0.46)	<.001
New Mexico	-0.52 (-2.19, 1.15)	0.542	0.31 (0.25, 0.38)	<.001
Washington	1.56 (-0.1, 3.22)	0.065	-0.71 (-0.74, -0.67)	<.001

Note that due to issues with non-parallel trends between state groups pre-2018 and due to Medicaid expansions, Louisiana and Utah were excluded from these analyses. States with relative increases in the proportion of early-stage diagnoses included California, Hawaii, New Jersey, and New Mexico; each of these states implement policies restricting STLD plans. Many of these states also implemented other policies that may be expected to improve access to care, but these policies differed by state with no other policies (i.e., not related to STLD plans) that were shared / commonly enacted between these states.

Supplementary Table 6: Falsification tests assessing changes in early-stage cancer diagnoses by STLD plan policies among individuals age ≥65 years

Analysis	State group	Temporal trends in % early stage (stages 0-II)		Unadjusted Analyses		Assessment of the parallel trends assumption				Adjusted Analyses			
		2016-2017	2019	Estimate (95% CI)	P-value	Main Analyses		Excluding states with Medicaid eligibility changes[a]		Main Analyses		Excluding states with Medicaid eligibility changes[a]	
						Estimate (95% CI)	P-value	Estimate[a] (95% CI)	P-value	Estimate (95% CI)	P-value	Estimate[a] (95% CI)	P-value
State-level STLD plan policies	No STLD plan policies[b]	59.2	59.2	Reference		Reference		Reference		Reference		Reference	
	Some STLD plan regulation[c]	59.9	59	-0.88 (-2.24, 0.48)	0.21	-1.5 (-2.62, -0.38)	0.009	-0.84 (-1.88, 0.2)	0.115	-0.74 (-1.99, 0.52)	0.249	-0.48 (-1.47, 0.52)	0.349
	Prohibit STLD plans[d]	59.2	58.9	-0.27 (-1.31, 0.76)	0.61	0.18 (-0.67, 1.03)	0.68	0.63 (0.05, 1.22)	0.033	0.13 (-0.81, 1.07)	0.786	-0.21 (-1.09, 0.68)	0.644
State-level STLD plan availability	STLD plans available[e]	59.2	59.1	Reference		Reference		Reference		Reference		Reference	
	No STLD plans available in 2019[f]	59.4	59	-0.35 (-1.38, 0.68)	0.51	0.12 (-0.75, 0.99)	0.786	0.71 (0.22, 1.19)	0.004	0.05 (-0.92, 1.02)	0.914	-0.01 (-0.78, 0.76)	0.979

[a]Excluded states include Louisiana (expanded Medicaid in mid-2016), Utah (partial Medicaid expansion in 2019), and Connecticut (multiple Medicaid eligibility changes/restrictions introduced from 2015-2018). See Supplementary Table 1 for additional information.

[b]Georgia, Iowa, Kentucky, and Utah.

[c]Connecticut, Hawaii, New Mexico, and Washington

[d]California and New Jersey.

[e]Georgia, Iowa, Kentucky, Louisiana, Utah, and Washington.

[f]California, New Jersey, and Hawaii. Note that New Mexico also introduced policies that ultimately led to insurers to stop offering STLD plans, but this occurred in mid-2019, so this state was excluded. See Supplementary Table 1 for additional information.

SUPPLEMENTARY FIGURES

Supplementary Figure 1: Derivation of the final analytic sample

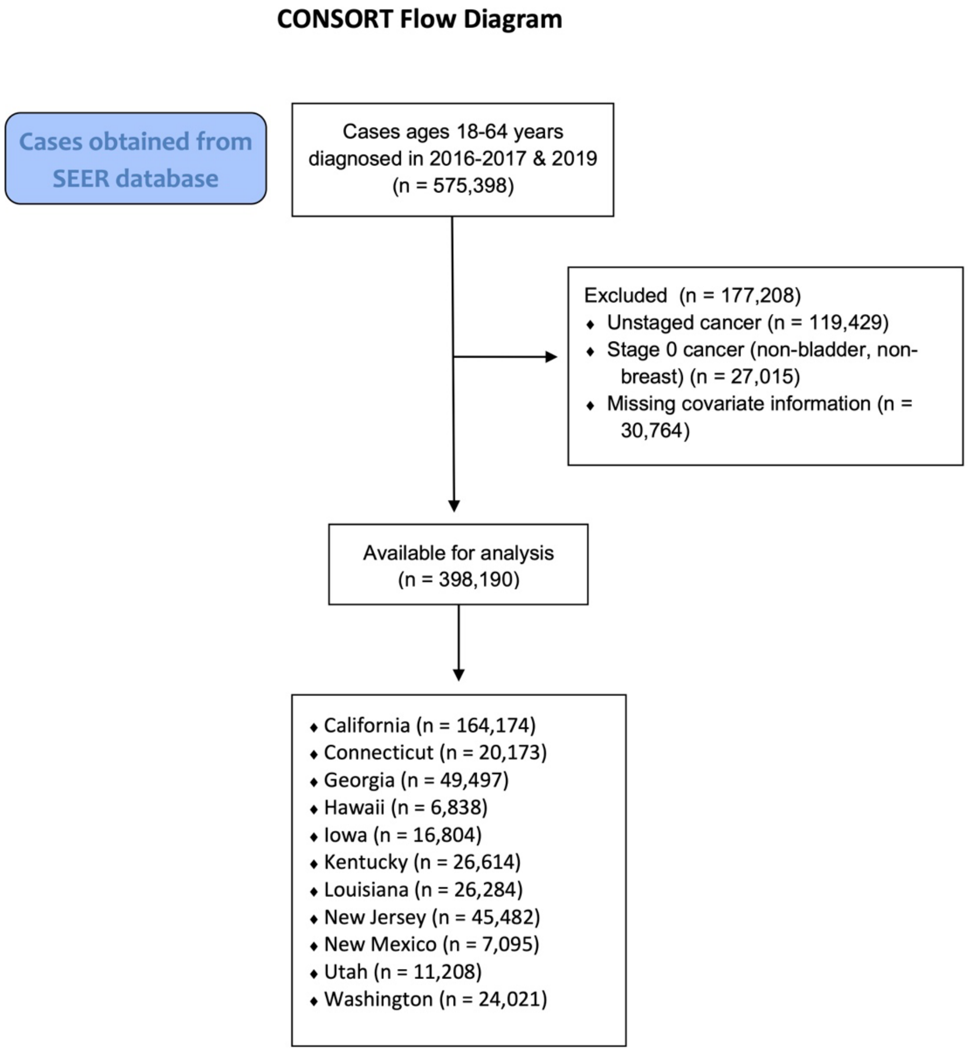

Legend: Note that unstaged cancers includes cancers for which no stage is applicable (e.g. central nervous system tumors, leukemia, and myeloma, which together consisted of 44,253 of the 119,429 cases). See supplementary methods for a sensitivity analysis where other unstaged cancers were included.
